# The Platform Switching Approach to Optimize Split Crest Technique

**DOI:** 10.1155/2014/850470

**Published:** 2014-08-06

**Authors:** G. Sammartino, V. Cerone, R. Gasparro, F. Riccitiello, O. Trosino

**Affiliations:** ^1^Unit of Oral Surgery and Implantology, University of Naples Federico II, Naples, Italy; ^2^Oral Surgery, University of Naples Federico II, Naples, Italy; ^3^University of Naples Federico II, Naples, Italy; ^4^Endodontics, University of Naples Federico II, Naples, Italy

## Abstract

The split crest technique is a reliable procedure used simultaneously in the implant positioning. In the literature some authors describe a secondary bone resorption as postoperative complication. The authors show how platform switching can be able to avoid secondary resorption as complication of split crest technique.

## 1. Introduction

Implant rehabilitation of edentulous sites with bone atrophy represents a situation in which dental implant placement might be complex or impossible if regeneration and bone augmentation techniques are not used [1]. One of the these techniques is ERE, introduced by Tatum [2] and subsequently modified by Scipioni et al. [3].

This procedure is mainly indicated in cases with sufficient bone height but inadequate thickness. Anyway at least 2-3 mm of initial crestal width is mandatory to perform this technique [4].

ERE can be followed by simultaneous implant placement in order to maintain the created space. This space can be filled with autologous/heterologous graft, with biomaterials or leaving the clot stabilized by a membrane [5–8], with or without the application of platelets concentrates such as PRP or PRF that seems to accelerate the healing of hard and soft tissues [9–11].

ERE surgical approach is a reliable technique but does not prevent the peri-implant bone resorption [12].

Different studies have evaluated the peri-implant bone resorption after implant positioning with ERE technique. Strietzel et al. report that 6 months after functional loading the marginal bone around the implants was reduced on average by 1 mm [13]. In another study, during the same period of observation, the mean bone loss was 2 mm [14]. Jensen et al. reported an average of resorption of 1.57 mm (mesial side) and 1.42 mm (distal side) during an average 4.2 years [15].

This case shows the absence of bone resorption and a slight bone apposition above the implant in a split crest technique using platform switching associated with Morse-cone connection.

## 2. Case Presentation

The patient was a healthy, nonsmoker, 26-year-old woman. Her dental history included an orthodontic treatment finalized in restoring the occlusion and repositioning the 47 that was tilted because of the loss of 46, extracted 10 years prior due to destructive caries.

**Figure 1:**
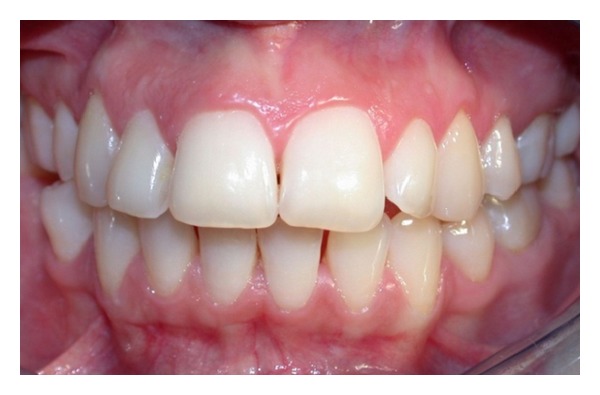
Frontal view.

**Figure 2:**
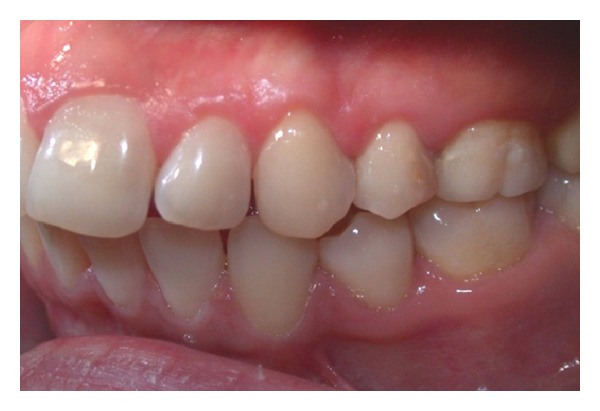
Lateral view (left).

**Figure 3:**
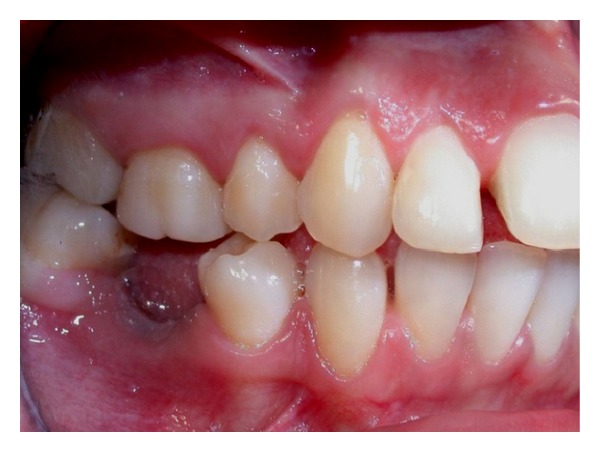
Lateral view (right).

**Figure 4:**
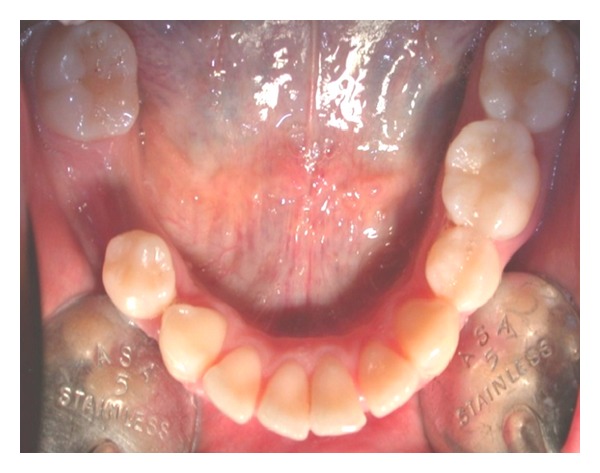
Occlusal view.

**Figure 5:**
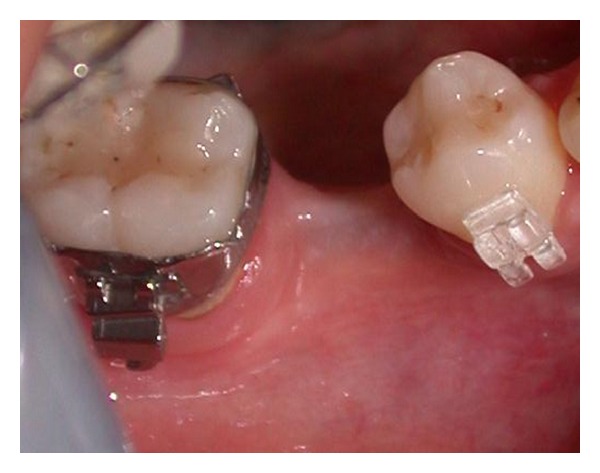
Site of 4.6.

**Figure 6:**
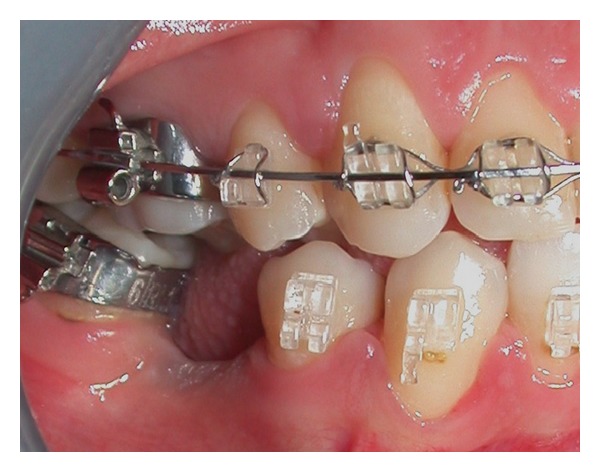
Site of 4.6.

Clinical examination (Figures 1, 2, 3, 4, 5, and 6) showed the lack of 4.6 and 1.5, 2.5, 3.5, and 4.5 in the arch; a ridge defect with reduction in bone thickness was diagnosed in region 4.6.

**Figure 7:**
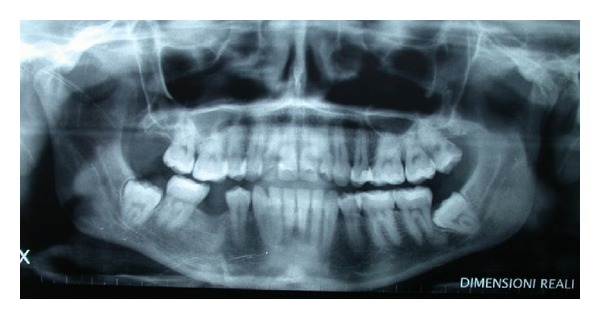
Panoramic view (OPT).

**Figure 8:**
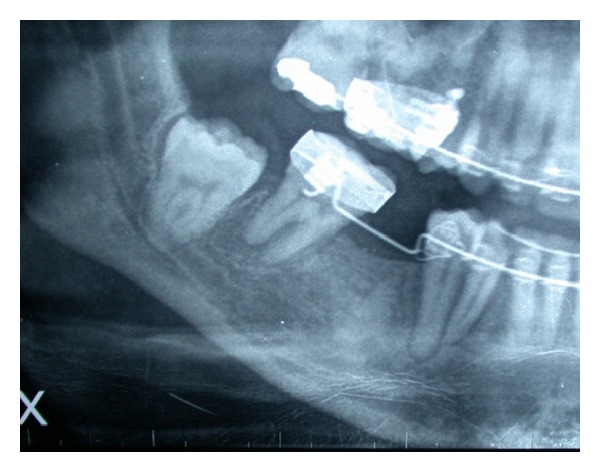
Radiographic view of 46.

**Figure 9:**
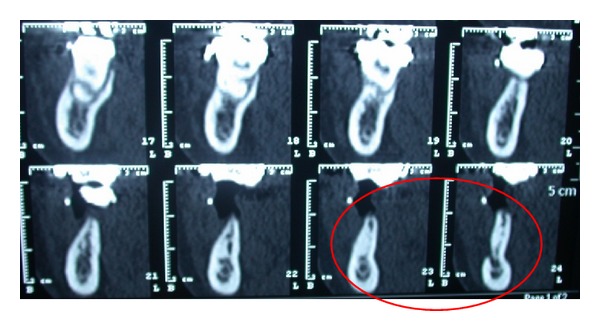
CT DentaScan.

OPT revealed the absence of second premolars and inclusion of third molars (Figures 7 and 8). A CT DentaScan tomography (Figure 9) showed 13 mm in ridge height and 3 mm in thickness of in the coronal segment of the ridge, classified as type 4 of Cawood and Howell.

In this condition, there is no indication for implantology if not preceded by ERE.

The patient followed premedication protocol: hygiene treatment and instructions during the days before surgery, antibiotics (2 g amoxicillin 60 minutes before the surgery), and 1 min rinse with chlorhexidine digluconate 0.12% preoperatively.

Peri-implant bone resorption was evaluated with a silicone gig using periapical radiographs that were taken on the day of the surgery and on the follow-up.

## 3. Surgical Procedures

Infiltrative anesthesia (mepivacaine 30 mg/mL and epinephrine 0.01 mg/mL) was performed on vestibular and lingual sides.

The procedure started with a midcrestal incision, extended from the distal surface of 44 to the mesial surface of 47 with two vertical releasing incisions that were extended into the vestibular side. A mucoperiosteal flap was lifted from the top of the bone ridge and then continued with a partial thickness flap in the vestibular fornix to obtain a mobile flap permitting a tension-free suture.

For osteotomies the Piezotome (Satelec) was used in mode 2.

**Figure 10:**
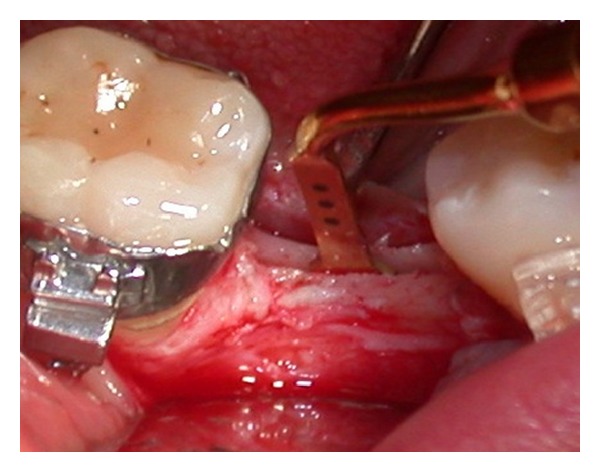
Midcrestal bone incision.

**Figure 11:**
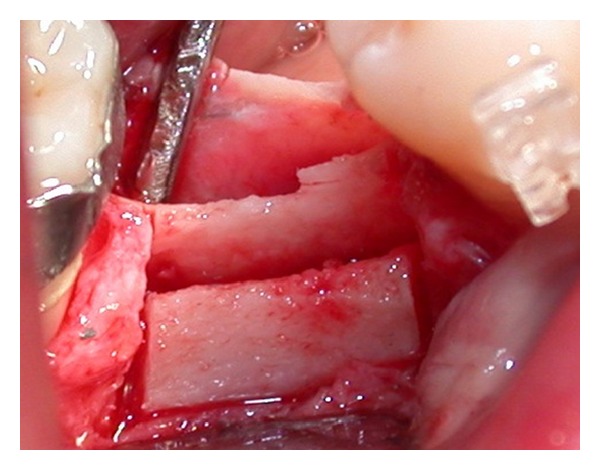
Longitudinal and vertical osteotomies.

The longitudinal bone crestal incision was performed and deepened down 6 mm (Figure 10). Subsequently vertical, mesial, and distal releasing incisions were performed from 1.0 to 1.5 mm away from the adjacent teeth. During this procedure, separation of the periosteum from bone was noted, probably due to the piezosurgery cavitation effect, which created a subperiosteal unstick mini emphysema (Figure 11).

**Figure 12:**
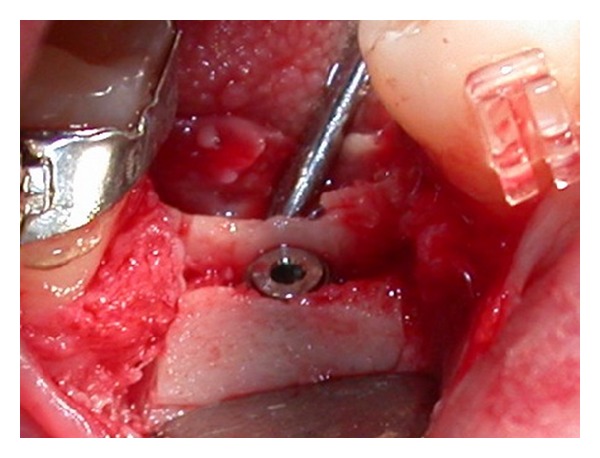
Clinical view after implant placement.

**Figure 13:**
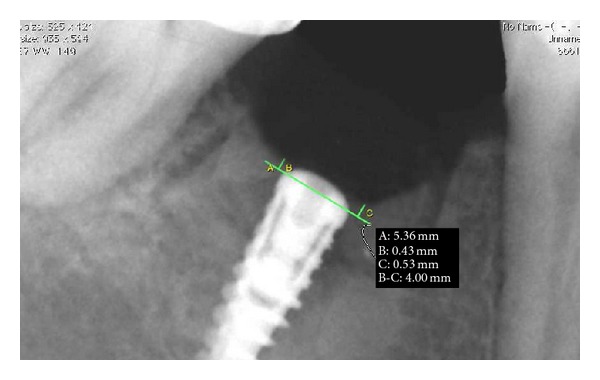
Immediate postoperative radiograph.

A progressive increasing diameters osteotomy from 1 mm to 3.5 mm was used to expand vestibular bone flap. After expansion, drills from 2 mm to final 3.5 mm diameter were used at 12.5 mm depth for implant site preparation. Implant was subcrestally placed (Figures 12 and 13).

One implant (In-Kone Universal, Tekka) 3.5 mm in diameter and 11.5 mm in length was placed.

Soft tissues were sutured without tension thanks to the partial thickness flap, and the implant was completely submerged. After the surgery, patient was encouraged to take, in case of pain, acetaminophen (1 g/8 hours) or ibuprofen (600 mg/8 hours).

After 4 months and half the second stage surgery was performed, making sure that the adherent gingiva surrounds the entire implant.

**Figure 14:**
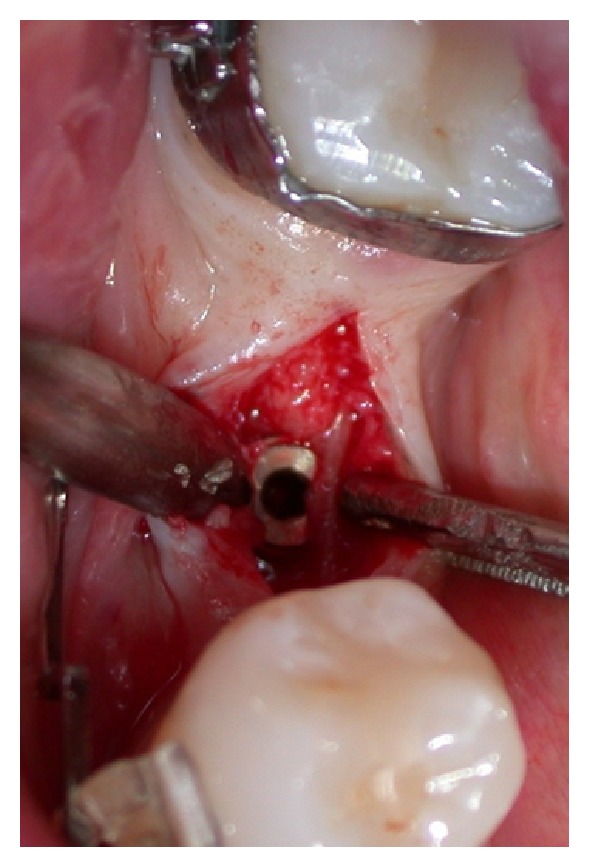
Healing abutment positioning.

**Figure 15:**
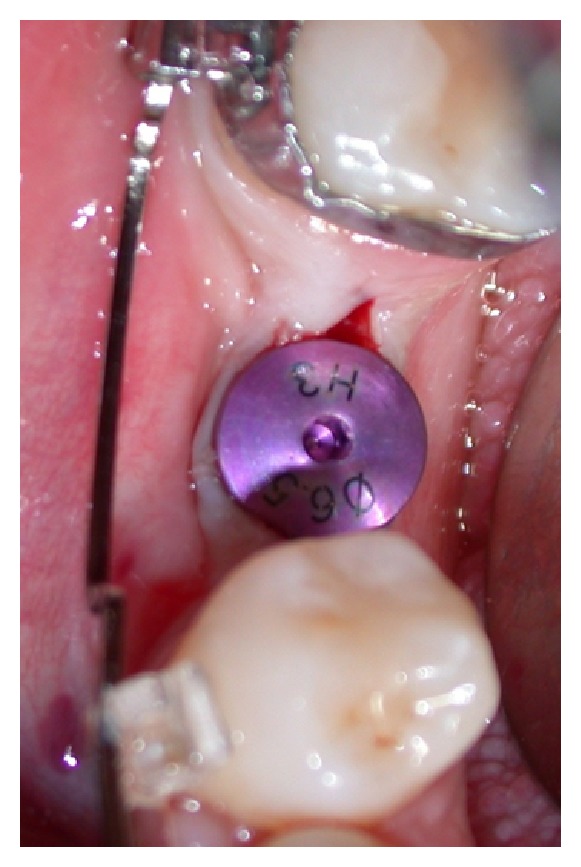
Healing abutment positioning.

**Figure 16:**
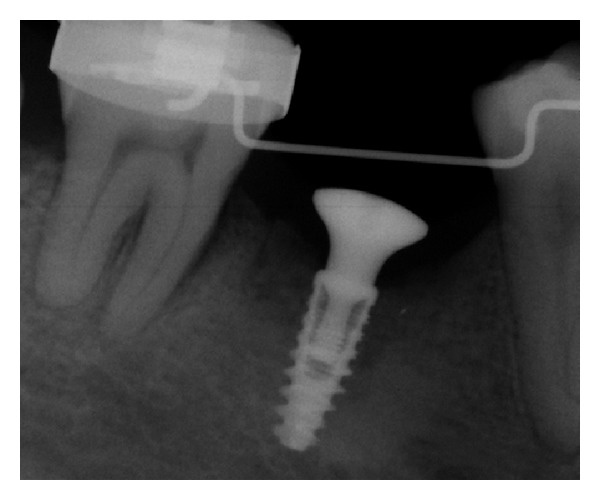
Radiographic control of the healing abutment adjustment.

Healing abutments were placed and an intraoral radiograph was performed (Figures 14, 15, and 16).

**Figure 17:**
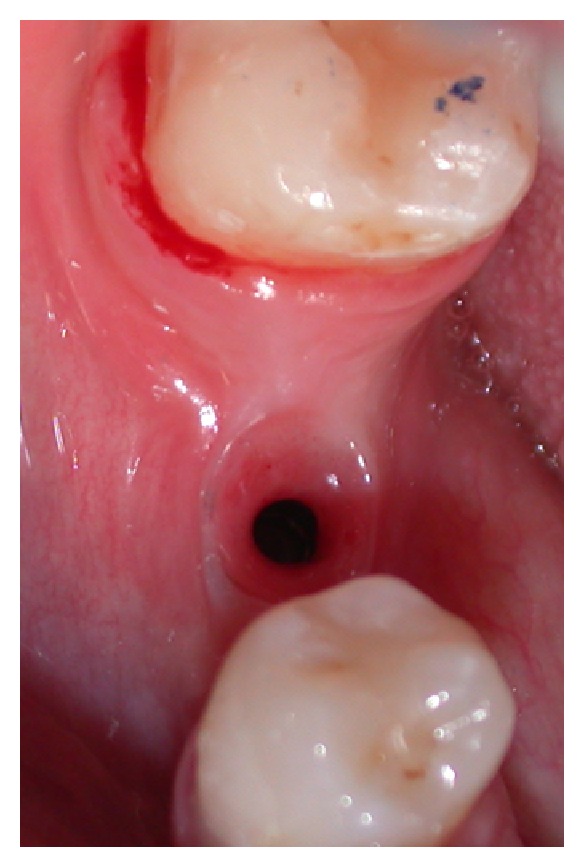
Provisional resin crown placement.

**Figure 18:**
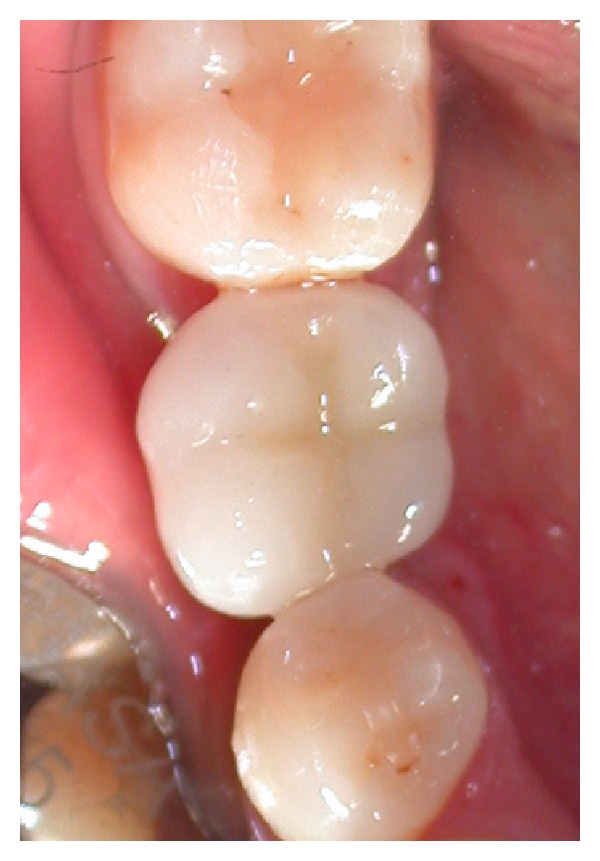
Provisional resin crown placement.

After 15 days a provisional resin crown restoration was positioned and maintained for two months in order to condition peri-implant soft tissue and optimize the emergency profile (Figures 17 and 18).

**Figure 19:**
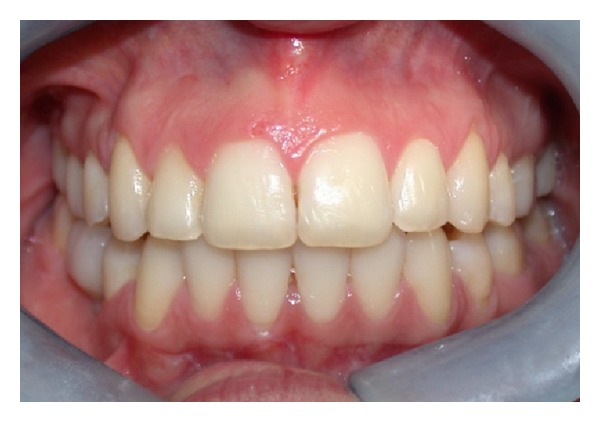
Clinical view of definitive crown.

**Figure 20:**
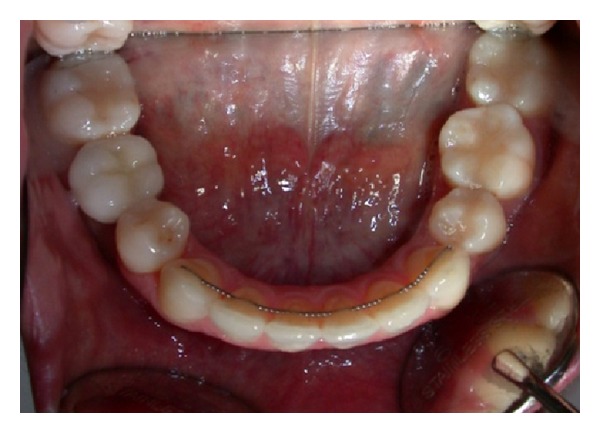
Clinical view of definitive crown.

After 60 days the definitive crown was placed (Figures 19 and 20).

**Figure 21:**
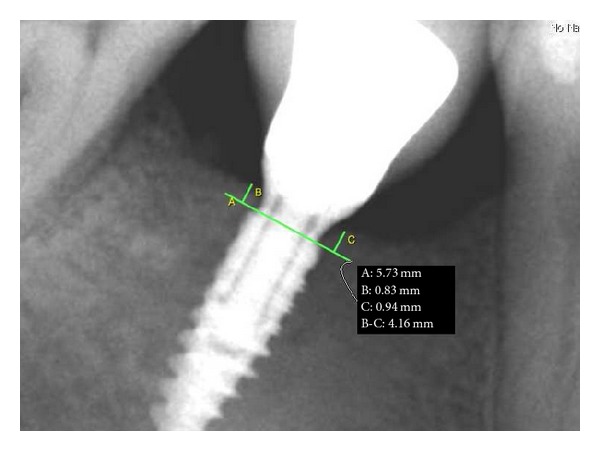
One-year radiographic follow-up.

Implant was controlled one year after loading. Clinical evaluation detects the absence of inflammation of the soft tissues, the absence of gingival recession, and stability of the prosthetic crown. An intraoral rx with silicone gig was performed. Bone level measurement was performed using digital program OsiriX and it was measured from the most coronal point of bone crest to the implant shoulder on the mesial and distal sides. Radiograph showed the absence of bone resorption and the bone growth above the implant shoulder; bone height was 0.83 mm on distal side and 0.94 mm on mesial side (Figure 21).

## 4. Discussion and Conclusion

Split crest procedure is mainly indicated in cases with presence of reduced crestal width and adequate height. Mandibular anatomical modifications following the postextraction resorption can complicate the implant placement and thus can be accompanied by several complications especially due to neurovascular lesions [16]. To avoid these complications it is necessary to perform a careful radiographic evaluation of mandibular anatomy. In the posterior region of the mandible attention should be paid to evaluate the distance to the inferior alveolar nerve in order to avoid neurosensory disturbance [17] as well as the angulation of implant placement to prevent the lingual cortical perforation and consequently the damage to the arteries of the mouth floor, mainly the mylohyoid artery [18]. In order to obtain an optimal implant primary stability, during split crest technique, it is necessary to place the implant apical to the osteotomic vertical lines, so greater attention needs to be paid to these structures.

In the literature crestal bone level around dental implants following restoration has been widely discussed. The factors that can explain the changes in bone height are gingival biotype [19], the distance of the implant-abutment junction (IAJ) from the bone crest [20], repositioning of the gingival inflammatory infiltrate, and the distribution of load in the portion of the implant in contact with the cortical bone [21]. Other secondary factors include the kind of surgical flap, second stage surgery for exposing screw [22], and colonization by bacteria belonging of the oral flora [23].

Also characteristics such as implant design (platform switching, Morse-cone connection, and rough shoulder) and the position of implant with respect to the bone crest may be involved in this process.

Platform switching can reduce crestal bone loss through four mechanisms [24]:

1. shifting of the inflammatory cell infiltrate inward and away from the adjacent crestal bone;
2. maintenance of biological width and increased distance of IAJ from the crestal bone level in the horizontal way;
3. reducing the possible influence of microgap on the crestal bone;
4. decreasing stress levels in the peri-implant bone.

In the studies on platform switching involving a follow-up period of 4–169 months, the reported bone loss varies between 0.05 and 1.4 mm [25].

Morse-cone connection determines zero microgaps and the absence of micromovement. Degidi et al. reported that, in presence of Morse-cone connection, platform switching shows no resorption [26].

Regarding the position of implant with respect to bone crest less resorption may be expected when implants are inserted from 1 to 2 mm subcrestally [27].

The results of this procedure can be improved thanks to some different implant macromorphologies, that is, matching the switching platform to the Morse-cone connection and presenting a nonsmooth collar, which maybe can allow bone growth on the implant shoulder.

However many other studies, including more patients, are necessary to confirm our result.

It will be interesting if this will be confirmed in order to optimize nontotally predictable bone augmentation techniques.
